# Therapeutic opportunities and clinical outcome measures in Duchenne muscular dystrophy

**DOI:** 10.1007/s10072-022-06085-w

**Published:** 2022-05-24

**Authors:** Giulia Ricci, Luca Bello, Francesca Torri, Erika Schirinzi, Elena Pegoraro, Gabriele Siciliano

**Affiliations:** 1 Department of Clinical and Experimental Medicine, University of Pisa, Pisa, Italy; 2 Department of Neurosciences, University of Padua, Padua, Italy

**Keywords:** Duchenne muscular dystrophy, Outcome measures, Therapy

## Abstract

**Introduction:**

Duchenne muscular dystrophy (DMD) is a devastatingly severe genetic muscle disease characterized by childhood-onset muscle weakness, leading to loss of motor function and premature death due to respiratory and cardiac insufficiency.

**Discussion:**

In the following three and half decades, DMD kept its paradigmatic role in the field of muscle diseases, with first systematic description of disease progression with ad hoc outcome measures and the first attempts at correcting the disease-causing gene defect by several molecular targets. Clinical trials are critical for developing and evaluating new treatments for DMD.

**Conclusions:**

In the last 20 years, research efforts converged in characterization of the disease mechanism and development of therapeutic strategies. Same effort needs to be dedicated to the development of outcome measures able to capture clinical benefit in clinical trials.

## Introduction: historical perspective

Duchenne muscular dystrophy (DMD) is devastatingly severe genetic muscle disease, which affects approximately 1:5000 ~ 10000 males [1] and causes childhood-onset muscle weakness and wasting, leading to loss of motor function and premature death due to respiratory and cardiac insufficiency [2]. Possibly due to its frequency and severity, DMD was the first genetic muscular disorder to be systematically described, starting with early reports such as those by Meryon and Conte, and then in the monographic works by Duchenne and Gowers (*circa* 1870) [3]. More than a century later, in 1987, DMD was again at the forefront of the neuromuscular field, as Eric P. Hoffman and Louis Kunkel demonstrated the absence of the protein dystrophin from the sarcolemma of DMD muscle fibers [4]. The lack of dystrophin was demonstrated to derive from mutations, mainly large rearrangements, in the *DMD* gene located on chromosome Xp21 [5]. These seminal discoveries marked the first time that the molecular bases of a genetic muscle disease were elucidated and had two important corollaries: first, that the murine disease observed in the *mdx* mouse was homologous to human DMD, thus establishing the first animal model of a genetic muscle disease; and second, that partial vs. complete dystrophin defects, caused by truncating vs. non-truncating *DMD* mutations, were responsible for DMD vs. the milder allelic dystrophinopathy known as Becker muscular dystrophy (BMD) [6].

In the following three and half decades, DMD kept its paradigmatic role in the field of muscle diseases, with the first clinical trials of glucocorticoids in the 1990s [7], the first systematic description of disease progression with ad hoc outcome measures [8, 9], and the first attempts at correcting the disease-causing gene defect by several molecular and gene-transfer approaches [10].

## Molecular basis of DMD

DMD is a progressive muscular dystrophy due to the absence of dystrophin protein in muscle cells because of mutations in *DMD* gene (Xp21.2-p21.1) disrupting the reading frame or by generating a premature stop codon. The dystrophin protein is a cytoskeletal protein that acts as shock absorber during muscle fiber contraction by linking the sarcomere actin to the extracellular matrix that surrounds muscle fibers trough the dystrophin-associated protein complex (DAPC). When this connection is lost, muscle fibers are damaged during contraction, thus leading to degeneration, secondary inflammation, and substitution of muscle by fat and fibrotic tissue.

DNA testing is crucial for DMD patients since it is important for diagnosis, genetic counseling, and family planning but also provides information on eligibility for mutation-specific treatment, as detailed below.

The *DMD* gene is the largest known human gene, containing 79 exons spanning 2.2 Mb. The mutation rate is high, since in one-third of cases, DMD is caused by a de novo mutation. The majority of patients have a deletion (∼68%) or duplication (∼11%) of one or more exons, while small mutations are involved in ∼20% of patients. These deletions and duplications are concentrated between exons 45–55 and exons 2–10, respectively. If the number of nucleotides of the exons deleted or duplicated is not divisible by 3, the reading frame is disrupted, leading to production of a truncated protein that does not contain the N-terminal and C-terminal domains crucial for connecting the actin to the extracellular matrix. In 20% of cases, small mutations including small deletions or insertions can disrupt the reading frame at an exon level. Point mutations can convert a codon for an amino acid into a stop codon or disrupt a splice site, thus also causing a premature termination of protein translation [10].

## DMD natural history

In DMD, muscle weakness begins as early as age 2 or 3, first affecting the proximal muscles, with progressive difficulty jumping, running, and walking. Other typical signs include enlargement of the calves, a waddling gait, and lumbar lordosis. The muscle wasting is rapidly progressive, and most patients use a wheelchair by their early teens. Weakness and musculoskeletal deformities as scoliosis result in early impaired pulmonary function, commonly around 20 years of age. Dilated cardiomyopathy is also part of the primary manifestation of disease. The disease history is also characterized by nutritional complications, including weight gain or loss, swallowing dysfunction with mandibular contracture and macroglossia, and in the advanced stages dysphagia. Endocrine complications, including impaired growth, delayed puberty, and adrenal insufficiency, are often reported and need to be monitored. Cognitive and/or behavioral issues can be presented in some boys, although are not progressive. Patients with DMD die between third and fourth decade of life for cardiac and/or respiratory failure. As standards of care for DMD have evolved, survival has also improved. Updated care considerations for DMD have recently been published, including recommendations on standard use of oral glucocorticoids, cardiac and pulmonary management, vaccinations, physical therapy, nutritional management, and bone health. The goals of the standards of care are to improve long-term survival, maintain mobility and independence, and improve quality of life [11–13]. Early diagnosis and an appropriate and multidisciplinary care provide the best opportunity for maximum benefit of both current standard and upcoming novel therapies (Table 1).

**Table 1:** Pharmacotherapy strategies in DMD. *AONs* antisense oligonucleotides

Treatment class	Mechanism of action	Molecule	Dosage	Currently in use/clinical trial phase
Steroids	Anti-inflammatory, anti-fibrotic action	Prednisone	0.75 mg/kg/d	Currently in use
		Deflazacort	0.9 mg/kg/d	Currently in use
		Vamorolone	2.0–6.0 mg/kg/d	Open-label, expanded access protocol ongoing
		Edasalonexent	100 mg/kg/d	Recruitment for open label phase terminated since phase 3 study did not meet the primary endpoint
AONs	Inhibition of exon inclusion into mature mRNA by the splicing machinery. Specific for some gene deletions	Drisapersen		Study withdrawn in 2016 for failing primary endpoint
		Eteplisersen		Approved by FDA, not by EMA
		Golodirsen		Approved by FDA, not by EMA
		Viltolarsen		Phase 3, open-label study ongoing
Stop-codon readthrough	Ribosomal readthrough; specific for nonsense mutations	Ataluren	10 + 10 + 20 mg/kg/d	Available; phase 4 study ongoing (STRIDE)
Gene replacement	Delivery of microdystrophin transgene via AAV to muscle cells to allow expression of functional protein	SRP-9001		Phase 2 study ongoing
Gene editing	CRISPR/Cas9-based gene editing technique aiming at restoring dystrophin expression in muscle cells and cardiomyocytes			Pre-clinical animal testing ongoing
Non-dystrophin restoring	Anti-oxidant, anti-inflammatory action	Idebenone		Phase 3 study ongoing
		Givinostat		Phase 3 study ongoing

## Pharmacological treatment: the present and the future perspectives

### Standard treatment

Therapy with oral glucocorticoids (GCs), including prednisone and deflazacort, an oxazoline derivative of prednisolone, represents the world-wide standard of care to date [14, 15]. Notably, new synthetic steroids, vamorolone and edasalonexent, are under development for the treatment of DMD [16].

The rationale to use steroids is based on complex and manifold mechanisms, including the inhibition of nuclear factor kappa-B and its downstream pro-inflammatory effects and other relevant pathways such as fiber-type transition from glycolytic to oxidative, widespread regulation of gene expression, membrane stabilization, stimulation of regeneration and repair, regulation of calcium metabolism, and possibly regulation of utrophin expression [17–20].

GC therapy is to date recommended for boys from 3 years old [15]. According to the 2018 consensus on diagnosis and management of Duchenne muscular dystrophy [11], “it’s never too late to start GCs in DMD,” as motor and general benefits can be observed at any age of therapy start. Notably, GC therapy should not be discontinued after loss of ambulation, given its efficacy also on non-motor features of the disease.

Standard dosage is 0.75 mg/kg/d for prednisone and 0.9 mg/kg/d for deflazacort (maximum daily dosage in patients weighing more than 40 kg are 30 mg prednisone and 36 mg deflazacort) [21]. The FOR-DMD (Finding the Optimal Regimen for Duchenne Muscular Dystrophy) trial has been specifically conceived to assess the level of standardization within the various regimens [22].

The standardized use of steroids treatment has changed over the years the natural history of DMD and, for this reason, is considered to date a prerequisite for enrollment in most clinical trials, improving outcomes by prolonging walking ability for 2–5 years, reducing the decline of upper limb strength and of respiratory and cardiac function, the need for scoliosis surgery [23].

More recently, vamorolone [24] has been proposed in DMD as a novel steroid analog based on membrane-stabilizing and anti-inflammatory properties (including inhibition of NF-kB) without significant immunosuppressive or hormonal effects [25]. A recent open-label, multiple-ascending-dose study provided Class IV evidence that for boys with DMD, vamorolone had possible efficacy compared to a natural history cohort of glucocorticoid-naive patients and appeared to be tolerated. Another proposed steroid for DMD has been edasalonexent, an inhibitor of NF-κB with anti-inflammatory properties [26]. A phase 2 study in DMD patients has recently been completed [27], with a phase 3 study currently being planned.

Overall, there are yet several open questions about the observed different individual response to GCs [28] and the management of the emerging disease phenotypes that are progressively becoming part of clinical practice given the modifications of disease natural history.

### Experimental treatments

#### Dystrophin-restoring approaches

The discovery that DMD is due to a defect of dystrophin [4] provided the rationale for a cure: if dystrophin expression was restored in muscle fibers, they would be preserved from degeneration.

The translation of dystrophin restoration to clinical practice, “from bench to bedside,” has been the object of enormous efforts over the last three decades. These may be broadly categorized under four main approaches, summarized below.

##### Molecular treatments (“exon skipping” and stop codon readthrough)

With the term “molecular treatments,” we indicate drugs that aim to obtain the expression of viable dystrophin protein from the patient’s own genomic material, by modulating RNA splicing (“exon skipping”) or mRNA translation (stop-codon readthrough) in such a way that completely or partially corrects the effect of the disease-causing mutation.

This strategy was made practically possible by the advent of antisense oligonucleotides (AONs). AONs are nucleotide oligomers (approximately 20–30 units), able to target and bind RNA in a sequence-specific fashion, but with a chemically modified backbone which confers resistance to nucleases and a pharmacokinetic and toxicity profile apt for human administration [29]. AONs for exon skipping are targeted at splicing enhancer sequences situated within or in close proximity of exons, thus inhibiting exon inclusion into mature mRNA by the splicing machinery. Clinical development plans were deployed, with AONs targeting several exons in the main mutational hotspot of the *DMD* gene, i.e., exons 44, 45, 51, and 53. Unfortunately, the road towards approval of exon-skipping AONs turned out less smooth than expected [30]. The company Biomarin-Prosensa launched a phase 3 trial of drisapersen [31], an AON drug based on the 2′-O-methyl chemistry and aimed at exon 51, which failed to demonstrate efficacy on the primary outcome, 6-min walking test (6MWT) distance, probably because of a combination of a narrow therapeutic window and challenges in clinical trial design [32]. Eteplirsen, also targeted to exon 51 but based on the PMO chemistry, was tested by the company Sarepta in a small cohort of 12 DMD patients with eligible deletions, who showed some degree of stabilization in ambulatory capacity and detectable dystrophin levels, although in the range of 1% of normal controls [33]. These data, especially those regarding dystrophin expression, led to an accelerated Food and Drug Administration (FDA) approval of eteplirsen in the USA [34]. The PMO golodirsen, targeted at exon 53 and developed by the same company, recently followed suit [35]. Conversely, the European Medicines Agency (EMA) denied such approvals on the grounds of lack of placebo-controlled clinical data [36]. Despite controversies [37, 38], exon skipping remains a very promising treatment approach for DMD patients with eligible mutations, and novel AON designs such as cell-penetrating, peptide-conjugated PMOs [39] might increase dystrophin-restoring potency and clinical efficacy.

A different mutation class amenable to molecular treatment is represented by nonsense mutations, which account for about 10–15% of DMD-causing mutations. The small molecule, orally administered drug ataluren, was developed by PTC Therapeutics with the explicit intent of retaining the same potential for ribosomal readthrough as aminoglycosides, but reduced toxicity. The drug underwent a series of clinical trials, with reassurances of safety in a first phase 2a trial [40], followed by controversial findings in a phase 2b trial which surprisingly demonstrated better efficacy with a lower (10 + 10 + 20 mg/kg/day) rather than higher (20 + 20 + 40 mg/kg/day) dose range [41], probably because of “bell-shaped” pharmacodynamics also observed in preclinical studies [42]. The 10 + 10 + 20 mg/kg/day dosing was brought on to a larger phase 3 study which fell short of its primary endpoint of a significant difference in 6MWT change after 48 weeks compared to placebo, in the *n* = 228 intent-to-treat population [43]. However, a pre-specified sub-analysis in participant with a baseline 6MWT distance between 300 and 400 m, known to predict a linear decline over the 1-year time frame of the study, showed a statistically significant 42.9 m difference in favor of ataluren, a finding also supported by evidence of delayed loss of motor function as assessed by several items of the North Star Ambulatory Assessment (NSAA) scale. These studies represented the basis for a conditional approval of ataluren by the EMA [41], with an indication restricted to ambulatory patients; however, the drug was not approved by the FDA. A large post-marketing registry (phase 4 study) of patients treated with ataluren worldwide, dubbed “Strategic Targeting of Registries and International Database of Excellence” (STRIDE) [42], is ongoing, with strong evidence of safety after years of administration and initial hints of long-term efficacy, i.e., delayed loss of ambulation [43].

##### Gene replacement treatments

Since the discovery of the molecular cause of DMD, the possibility to obtain a definitive cure through the delivery of a functional copy of the *DMD* gene has been obvious. However, to allow that, a great research effort has been performed for the last decades. For single‐gene diseases resulting from absent protein expression, as in DMD, the objective of gene replacement therapy is to deliver an intact copy of the mutated gene, named transgene, to muscle cells to allow the expression of the functional protein and then to restore or attenuate the phenotype. In recent years, the use of adeno-associated viral (AAV) vectors has provided the possibility to deliver genetic material to a variety of tissues based on the tropism of different AAV serotypes. A synthetic microdystrophin has been designed in order to produce a short transgene that can allow the synthesis of a functional “in-frame” truncated protein as observed in Becker muscular dystrophy‐like condition. To date, only preliminary data are available. An open‐label phase 1/2a trial with microdystrophin in four DMD children showed high levels of microdystrophin expression after three months from systemic AAV administration, in association with a clinical improvement. A double‐blind, placebo‐controlled trial is ongoing (NCT03769116).

##### Gene editing strategies

Among new therapeutic options for DMD, the CRISPR/Cas9-based gene editing technique is one of the promising future possibilities that are at the moment under development, aiming at restoring dystrophin level in muscle cells.

Recent experimental evidence has, in fact, shown a positive effect of dystrophin restoration not only on muscle cells but also on oxidative stress regulation and cell proliferation also in muscle stem cells. The technique consists of various means (AAVs, lentiviruses, electroporation, nucleofection) of delivering the Cas9 endonuclease along with a single-guide RNA; as the endonuclease acts on DNA producing double-strand break at the sequence level for which the RNA is designed for, the genomic sequence is then repaired via non-homologous end-joining (NHEJ) or homology-directed repair (HDR). On a theoretical basis, the first approach is more error prone, but it is prevalent in frequency in post-mitotic cells as a genetic repair mechanism; moreover, if applied to deletions or point mutations, it leads to a partially functioning protein. HDR, instead, is more precise, but a genetic-defect specific donor DNA is needed, and it is a rarer DNA-repair mechanism, thus complicating the engineering and delivery. Notably, this approach can be used to correct duplications, thus restoring a completely functional protein. A variant version of CRISPR-Cas9, CRISPRa, is under development, aiming at regulating transcription of compensatory proteins as LAMA1, utrophin, and Klotho. Pre-clinical trials on mice and canine models have shown restoration and expression of dystrophin in skeletal muscle and cardiomyocytes and increased survival in mice.

##### Non-dystrophin-restoring approaches

A number of non-dystrophin-restoring therapeutic approaches are under development, based on the complex histopathological and molecular mechanisms that are involved in DMD’s physiopathology, including inflammation, fibrosis, oxidative stress, intracellular homeostasis, and signaling. However, to date, none of these molecules have shown striking or particularly promising results in clinical trials, currently at phases II and III in the EU and the USA.

Among them, a known and recognized causative factor of muscle tissue depletion in DMD is fibrosis led by chronic inflammation. In response to this, the molecule Givinostat has been developed. Givinostat is an inhibitor of histone deacetylase (HDAC), which is upregulated in DMD muscle [44]. Evidence of the anti-inflammatory power of iHDACs via suppression of cytokines comes from various pre-clinical studies that opened the path for Givinostat to be tested in many clinical settings, from muscle dystrophy to diabetes and hematological diseases. The positive effect of Givinostat in DMD ambulant boys was firstly described in 2016 [45], and specifically, homogeneous increase of muscle fiber size was reported, associated with reduction of necrosis, fibrosis, and fatty infiltration, although no functional effectiveness measurement was made. At the moment, a phase III and long-term clinical trials are ongoing and recruiting also adult patients; no data is available at the moment about clinical results.

## Towards new therapies: proof of concept of trial readiness and outcome measure

More generally, the great phenotypic heterogeneity represents a critical issue in tracing clinical course of NMD, often associated to a lack of reliable biological and clinical markers which could be sensitive over-time in the disease progression and responsiveness to treatment. Related to this, one of the major critical issues to ensure the success of a clinical trial is an adequate targeting and stratification of the study population which, in turn, influences the set-up of outcome measures [46].

Although new appearing gene therapies in preclinical studies have proved increasingly feasible for long‐term safety and efficacy, in DMD, the enormous size of the gene, the presence of numerous isoforms expressed in muscle and non-muscle tissues, the large volume of muscle tissue in the human body involved represent a significative obstacle in focusing the therapeutic targets. In keeping with these findings, use of systems which allow capturing and matching genetic and clinical data is crucial to define trial feasibility and, beyond this, to trace the trajectory of the natural history of the disease and care monitoring. Clinical goals are to determine if data are available and sufficient to define the relationship between outcome measures and stages of disease progression. For that, the accuracy of patients’ phenotypic stratification is mandatory to clearly define disease and existing gaps [47].

In general, the outcome measures used in clinical practice can be divided into four categories: self-reported measures, performance-based measures, observer-reported measures (completed by a parent, caregiver), and clinician-reported measures (Table 2).

**Table 2:** Commonly used clinical outcome measures

Outcome measure	Brief description
6MWT	Patient walks for 6 min along a 25-m linear path. Total distance, interlap time, and need to stop or pause are recorded
NSAA	Quantitative functional scale for DMD/BMD ambulant patients
MFM	Quantitative scale that measures motor functional abilities in a person with neuromuscular disease
Timed motor tests (time to sit, time to stand, time to run 10 m, time to climb and descend 4 steps)	Time to complete the task is recorded
Handgrip strength	Vigorimeter measured handgrip strength
PedsQoL	Health-related quality of life assessing scale for patients aged 8–12 and parents
Biomarkers (muscle MRI, dystrophin level measurement, others)	

Functional outcome measures for evaluating patients with DMD in clinical trials have traditionally consisted of timed tests and motor scales as the 6-min walking test (6MWT), other timed tasks (time to stand, time to run 10 m, and time to climb and descend four stairs), the North Star Ambulatory Assessment (NSAA) scale, the Motor Function Measure (MFM) scale, grip-strength assessment, other clinical parameters as bone mass and BMI (especially in trials regarding steroid treatment), and cardiologic and respiratory parameters.

The NSAA is a 17-item scale with score ranging 0–34 evaluating motor tasks in ambulatory DMD patients, such as head raise, possibility to stand from lying on the floor and tasks evaluating the inferior limbs which are needed to define a patient functionally ambulant. Scores assigned to each task range from 0 (not able to perform the task) to 2 (complete ability to perform the task without external help). It also includes timed motor tests as the 10-m walk/run test, which has been demonstrated to predict age at ambulation loss [48]. This scale demonstrates a high intra- and interobserver reliability and is commonly included in DMD clinical trials.

The 6MWT is a timed motor test in which the patient is asked to walk along a 25-m track for up to 6 min; total walking distance, inter-lap walking time, and need to pause or stop the test are recorded and evaluated through normative data [49]. This test has been used in many neuromuscular diseases follow-up and clinical trials, including DMD, and can be particularly useful because it describes a parameter that can be translated in the global estimation of living function and quality of life. Mazzone et al. [50] in 2010 tested the correlation between NSAA, 6MWT, and other timed test performances in a cohort of 114 DMD boys and found a good correlation between scores obtained in NSAA and 6MWT, thus supporting the use of both these tests in longitudinal follow-up and clinical trials.

Other timed motor tests, such as the time to stand, time to run 10 m, and time to climb and descend four stairs, are easy to perform and feasible for a clinical setting, although showing less test–retest reliability compared to 6MWT [48], that on the other hand appears to be more strongly correlated to timed motor test performance than to quantitative strength measures.

The Motor Function Measure (MFM), including the Vignos functional grade, consists of a battery of motor tasks that patients are asked to perform and is suitable for nearly any age of examination, from young children to adult patients; it is used in many clinical trials for neuromuscular diseases and in current neurological examination. Joint contractures are also considered in this scale. It is particularly useful in the assessment of functionality of patients, which can correlate to the subject’s capability of performing daily-life activities.

At last, grip strength measurement had been widely used as an outcome measure in neuromuscular diseases clinical trials and assessment, although it requires a good level of patient comprehension and compliance and is not suitable for the youngest subjects.

Patients’ advocacy and scientific societies are increasingly asking for quality-of-life assessment as an outcome measure in clinical trials, which could indeed represent a reliable parameter to consider, if elaborated properly, especially in those patients already greatly compromised in which most of the motor tasks cannot be evaluated.

A commonly used scale is the PedsQoL for pediatric patients, and recently the proposal of a Clinical Global Impression of Change in DMD boys has been made by Staunton [51] based on interviews with clinicians and caregivers, with the aim of highlighting which variations in disease they valued as more meaningful, as sense of fatigue, endurance, and quality of movement (i.e., less toe-walking).

As highlighted by Merlini et al. [52], a first limitation to the cited motor tests in DMD is that they require a certain level of compliance and comprehension of the task, which in younger DMD patients may not be easily obtained, especially considering that hopefully diagnosis will be reached earlier and earlier in present and future times. Given the progressive course of the disease and the precocious start of muscle degeneration in patients that otherwise may not be included in clinical trials until the age of 6 or 7, preservation or progression rate decline would be more suitable as endpoint measures in clinical trials compared to gross motor tasks focused on muscle strength, such as the 6MWT, that may fail to show improvement or stabilization over the course of the observation period of the trial or, even worse, could show a transient improvement followed by decline. On the other hand, efficacy of early, long-term corticosteroid therapy in prolonging ambulatory period in some cases up to 16 years of age, which is considered the clinical boundary between DMD and BMD, poses as a reasonable parameter of efficacy for new therapeutic strategies of the achievement of this result at least before the age of 12, configuring an intermediate mild DMD-severe BMD phenotype.

Also, if diagnosis is made in a time when the clinical picture is not completely florid, such scales may not significantly capture improvement or stabilization of disease course. In this context, motor functional scales used in the assessment of other diseases such as the CHOP scale for SMA I-II, designed for little patients receiving an early treatment, could be a possible alternative. Another element to take into account is the clinical variability and stratification of phenotypes to which steroid treatment is leading DMD patients — as to date, the vast majority of clinical trials require steroid treatment as an inclusion criterion in order to standardize patients’ recruitment — characterized by intermediate DMD-BMD clinical pictures along with variable cardiac involvement, differently affecting motor performance. In the perspective of precocious diagnosis, screening programs, and early treatment start, other clinical outcome measures may be required, in order to assess treatment efficacy even in the youngest patients, among steroid-treated ones, and in severely compromised patients, paralleled by a refined phenotypic definition.

### The application of new eHealth technologies in disease monitoring: search for digital clinical biomarkers

A digital biomarker can continuously measure in objective manner functional motor parameters through digital biosensors, also in real-life setting. Especially in the pandemic COVID-19 scenario, the development of digital biomarkers appears crucial in the medical field. However, there is still little experience in applying digital biomarkers in clinical practice.

A recent study [53] performed a pilot study in seven non-ambulant DMD patients to demonstrate the feasibility and reliability of physical data recorded with a magneto-inertial sensor, ActiMyo containing a three-axis accelerometer, a three-axis gyroscope, and a three-axis magnetometer. This study demonstrated that the ActiMyo variables are able to be representative of movements performed during the tasks and correlated well with the scores obtained using validated tests, showing as potential good candidates of outcome measures in non-ambulatory DMD patients.

A subsequent study of Lilien et al. [54] explored the digital biomarker in home-based monitoring using a wearable magneto-inertial sensor (VMIS) for 23 ambulant DMD patients. The study demonstrated that the device’s variables were correlated with the clinical validated scores and are sensitive to change in the DMD patients over 6 months, thus providing objective and reliable data.

The authors of present review FT, GR, and GS (University of Pisa, unpublished data) are involved in a bioengineer project working on definition of an integrated, multiparametric approach by using a single software platform. Modules including neurological examination and functional motor tests, in addition to other clinical data from muscular MRI, genetic data, muscle biopsies, are under development and optimization to be used for phenotypic characterization and follow-up in muscular dystrophies.

## Conclusions

We are now experiencing a period of several therapeutic challenges in modifying the natural history of DMD. Among them, the more promising are the genetically mediated treatments as new disease-specific therapeutic options to attenuate the disease severity.

To find the most suitable outcome measure for clinical trial in DMD is still a hard work. Reasons for that include the relatively wide phenotypic variability, the different progression rate of each clinical variable indicative of muscle wasting progression, and at the moment limited expectation of the effects of therapeutic interventions, either pharmacological or not, on the natural history of the disease. Different pathways behind muscle damage repair for each therapeutic approach require a finely tuned selection of the appropriate instrument for detecting efficacy. Advancements of techniques, accurate but at the same time smart and not intrusive, are able to measure patient coping to disease burden and quality of life impairment in everyday activities and will drive the development of the field in the next years. This will give a great opportunity to improve availability of more confident measure indexes, reduce methodological biases, and overall enable the observer to make visible what has not to be invisible in clinical trials for DMD.
